# Supplementary material for: A UMLS-based spell checker for natural language processing in vaccine safety
Source: BMC Med Inform Decis Mak. 2007 Feb 12;7:3. doi: 10.1186/1472-6947-7-3 (PMC1805499; doi:10.1186/1472-6947-7-3)
Supplement: Additional file 13 — RAP application directory. Sets up directory for RAP (RDF application for PHP) source code [file 1472-6947-7-3-S13.gz › rap/doc/phpdoc/rdql/RdqlParser.html]

xml version="1.0" encoding="iso-8859-1"?


Docs For Class RdqlParser


## Class RdqlParser

Description

Description |
Methods (details)

This class contains methods for parsing an Rdql query string into PHP variables.

The output of the RdqlParser is an array with variables and constraints of each query clause (Select, From, Where, And, Using). To perform an RDQL query this array has to be passed to the RdqlEngine.

History:

- access: public
- author: Radoslaw Oldakowski <radol@gmx.de>
- version: V0.9.1

Located in /rdql/RdqlParser.php (line 35)

```
Object
   |
   --RdqlParser
```

Method Summary

Description |
Methods (details)

array
&parseQuery
(string $queryString)

Methods

Description |
Methods (details)

parseQuery (line 92)

Parse the given RDQL query string and return an array with query variables and constraints.

- access: public

array

&parseQuery
(string $queryString)

- string
  $queryString

#### Inherited Methods


Inherited From Object

> Object::toString()

Documentation generated on Thu, 7 Jul 2005 13:43:26 +0200 by phpDocumentor 1.3.0RC3
